# Antibacterial Potential of *Vatica diospyroides* Flower Extracts: Targeting Diverse Pathogens in Aquaculture

**DOI:** 10.1155/ijm/7471536

**Published:** 2025-06-30

**Authors:** Juthatip Yooklaen, Theera Srisawat, Luu Tang Phuc Khang, Nguyen Dinh-Hung, Papungkorn Sangsawad, Nguyen Vu Linh, Patima Permpoonpattana

**Affiliations:** ^1^Department of Agricultural Science and Technology, Faculty of Science and Industrial Technology, Prince of Songkla University, Surat Thani Campus, Surat Thani, Thailand; ^2^Department of Agricultural Science and Technology, Faculty of Innovative Agriculture, Fisheries and Food, Prince of Songkla University, Surat Thani Campus, Surat Thani, Thailand; ^3^Department of Animal and Aquatic Sciences, Chiang Mai University, Chiang Mai, Thailand; ^4^School of Animal & Comparative Biomedical Sciences, The University of Arizona, Tucson, USA; ^5^School of Animal Technology and Innovation, Suranaree University of Technology, Nakhon Ratchasima, Thailand

**Keywords:** acetone, antibacterial, flow cytometry, pathogens, *Vatica diospyroides*

## Abstract

*Vatica diospyroides*, an endemic species of the Dipterocarpaceae family, possesses notable medicinal properties. However, its application as an antibacterial agent is limited due to the insufficient investigations of its antibacterial activity from flower extracts. This study is aimed at exploring the antibacterial mechanisms of acetone extracts from the flowers of *V. diospyroides* against four bacterial strains using various methods, including the well-disk diffusion assay, minimum inhibitory concentration (MIC) determination, minimum bactericidal concentration (MBC) assessment, flow cytometry, and scanning electron microscopy. The inhibition zones measured between 6.33 and 17.66 mm. Notably, the extract exhibited different MIC values, such as 250 *μ*g mL^−1^ for *Bacillus subtilis* and *Escherichia coli*, and only 62.5 *μ*g.mL^−1^ for *Vibrio parahaemolyticus*, demonstrating its effectiveness. MBC values ranged from 500 to over 1000 *μ*g mL^−1^ for *Pseudomonas aeruginosa*. Flow cytometric analysis revealed that the cellular responses to the extract were influenced by both the concentration of the extract and the duration of exposure, indicating a dose- and time-dependent antibacterial effect. Additionally, scanning electron microscopy confirmed that the extract caused structural damage to the cells of both Gram-positive and Gram-negative bacteria. Overall, this study underscores the promising antibacterial potential of *V. diospyroides* flower extracts, which demonstrate significant efficacy against a variety of bacterial strains.

## 1. Introduction

Aquaculture, the rearing of aquatic animals and plants for food, is a very complex area with many variables [1]. The rapid development, intensification, and globalization of the sector have led to significant challenges, in particular the emergence and spread of diseases. This has led to a heavy reliance on antimicrobials to maintain production levels [2, 3]. As the treatment of individual animals in aquaculture is impractical, the metaphylactic use of antibiotics to treat entire populations has become standard practice. However, any exposure to antimicrobials—whether at therapeutic, chronic, or subtherapeutic doses—leads to the selection of resistant mutants that can arise spontaneously [1]. Therefore, there is an urgent need to research and develop new antibacterial agents that can reduce the use of antibiotics and combat the growing threat of antibiotic resistance [4]. This has driven research into the isolation and identification of plant-derived bioactive compounds with antibacterial properties [5]. Numerous plant species produce bioactive compounds, and their potential as antibacterial agents has been exploited in various contexts [6, 7]. However, a comprehensive understanding of these compounds is still lacking. As the antibacterial effects of many medicinal plants are still largely unexplored, researchers are increasingly focusing on the discovery of new, effective treatments [8, 9].

*Vatica diospyroides* Symington (VDS), commonly known as chan-ka-pho in Thai, is a medicinal plant valued for its rich phytochemical content (e.g., saponins, cardiac glycosides, flavonoids, tannins, terpenoids, and the resveratrol tetramer) [10–12]. Previous studies have identified two resveratrol derivatives, vaticaphenol and vaticanol, which play an important role in cardiovascular therapy and exhibit toxicity against various human cancer cell lines [11, 13]. Acetone and methanol extracts from VDS leaves and stems containing saponins, anthraquinones, and terpenoids offer numerous health benefits, including anti-inflammatory, anticancer, and cell-protective properties [12]. Additionally, extracts from the cotyledons and pericarps of VDS have been reported to induce antiproliferative and apoptosis-inducing effects in MDA-MB-231, MDA-MB-468, and MCF-7 breast cancer cells [14, 15]. This species contains mainly phenolic compounds that are widely used for their antibacterial properties [16]. These compounds exhibit different mechanisms of action against microbial strains, including an EP inhibitory effect, alteration of cell membrane permeability, disruption of intracellular functions by binding to enzymes, and impairment of cell wall integrity through interactions with the cell membrane [17–19]. Flavonoids from this plant have shown promising activity against *Escherichia coli*, *Pseudomonas aeruginosa*, *Klebsiella pneumoniae*, and *Mycobacterium tuberculosis* [20, 21]. Tannins, which are known for their antibacterial activity, attack both Gram-negative and Gram-positive bacteria by destroying the cell wall and membrane, inhibiting oxidative phosphorylation, altering microbial metabolism, intercalating into DNA base pairs, and disrupting microbial enzymes that suppress transcription and ultimately lead to cell death [22]. Previous studies on VDS, such as that of [23], have investigated the antibacterial activity of fruit extracts against both Gram-positive and Gram-negative bacteria. Their results showed that *Bacillus subtilis* responded to cotyledon and pericarp extracts in a dose-dependent manner, while *Staphylococcus aureus* responded to pericarp extracts in a time-dependent manner. Furthermore, Musimun et al. [24] demonstrated the synergistic antibacterial effect of silver nanoparticles (AgNPs) from *Phyllanthus emblica* in combination with VDS cotyledon extract. They found that the fractional inhibitory concentration index (FICI) showed synergistic activity against *S. aureus*, although no effect on *B. subtilis* was observed. Flow cytometry (FCM) showed that the proportion of *S. aureus* that responded to the combined treatment increased in a dose-dependent manner.

Although VDS is recognized as a potent medicinal plant, the antibacterial properties of its flowers, in particular, have not been thoroughly investigated. This study aims to explore the antibacterial activity of acetone extracts from the flowers of *V. diospyroides* by determining the minimum inhibitory concentration (MIC) and minimum bactericidal concentration (MBC) against common bacterial strains, evaluating the bacterial survival rates and reaction patterns by FCM, and analyzing the bacterial morphological changes by scanning electron microscopy (SEM).

## 2. Materials and Methods

### 2.1. Plant Materials and Bacterial Strains

The flowers of *V. diospyroides* were collected in Khian Sa district, Surat Thani province, Thailand. The samples were air-dried at room temperature (25 ± 1°C) and stored without direct sunlight until further experiments. The plant voucher Specimen No.: 1457-PSU was deposited in the Herbarium of Queen Sirikit Botanic Garden (QBG), Maerim, Chiang Mai, Thailand. All methods were carried out in accordance with relevant guidelines and legislation for plant-based studies.

**Figure 1:**
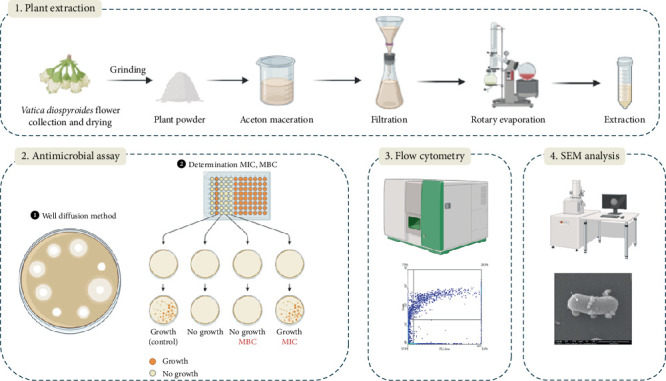
Schematic diagram of experimental design of this study.

Four bacterial strains, including *S. aureus* ATCC 25923, *B. subtilis* ATCC 6633, *E. coli* ATCC 25922, and *Vibrio harveyi* ATCC33867 were obtained and maintained at the Scientific Laboratory and Equipment Center of Prince of Songkla University, Surat Thani campus, Thailand. All the chemicals used in this study were of analytical grade and high purity. A schematic diagram of the experimental design is presented in Figure 1.

### 2.2. Preparation of *V. diospyroides* Flower Extract

*V. diospyroides* flower extraction (VFE) was performed using a modified version of the method described by Srisawat et al. [12]. In brief, 200 g of dried flower pieces were macerated in 500 mL of acetone (CH_₃_)_₂_CO (*w*/*v*) for 5 days at room temperature. The solvent was then evaporated to obtain the crude extract. The resulting extracts were then dissolved in 10% dimethyl sulfoxide (DMSO) to reach a final concentration of 20 ^−1^mg/mL. The VFE was stored in the dark at 4°C for future experiments.

### 2.3. Antibacterial Effect by Well Diffusion Assay

The antimicrobial activity of VFE was evaluated using an agar well diffusion assay according to the method described by Musimun et al. [24]. Approximately 100 *μ*L of a freshly grown bacterial culture (10^8^ CFU.mL^−1^) of freshly grown test bacterial culture was spread on Mueller–Hinton agar plates with the nontoxic swab. Wells with a diameter of 6 mm were punched into the agar using a sterile cork borer. Then, 25 *μ*L of VFE at different concentrations (10, 25, 50, 100, 200, 400, 600, 800, and 1000 *μ*g.disc^−1^) were aseptically added to each well using a micropipette. Ciprofloxacin (30 *μ*g disc^−1^) was used as a positive control for *S. aureus* and *B. subtilis* and oxytetracycline (30 *μ*g disc^−1^) as a positive control for *V. harveyi*. The inoculated plates were left for 1 h to allow the extract to prediffuse into the medium and then incubated at 37 ± 2°C for 18 h. The zones of bacterial growth inhibition were observed, and the diameters of these zones were measured in triplicate.

### 2.4. Determination of MIC and MBC

The MICs of VFE against the tested bacteria were determined using the broth microdilution method and the resazurin-based 96-well microdilution method according to the procedure described by Sarker et al. [25]. Stock solutions of VFE were prepared at a concentration of 2000 *μ*g.mL^−1^ in 1.5-mL Eppendorf tubes. Serial dilutions of the stock solution were prepared in the Mueller–Hinton broth (Becton Dickinson, Sparks, MD, United States) in 96-well microplates at concentrations ranging from 31,000 to 1.95 *μ*g mL^−1^. A bacterial suspension of approximately 10^8^ CFU/mL^−1^ was prepared from a 24-h culture plate. Then, 1000 *μ*L of this suspension were inoculated into each well. A sterility and growth control well were also studied for each strain. The microtiter plates were incubated at 37°C for 24 h. Wells 11 and 12 had negative (10% DMSO) and positive (3.5% EtOH) controls, respectively. A 10 mL of 6.75 mg/mL^−1^ resazurin solution was added to each well. After incubation, the MIC was defined as the lowest concentration at which the resazurin color remained blue, indicating no bacterial growth.

The MBC was defined as the lowest extract concentration that killed 99.9% of the bacterial inoculum after 24 h of incubation at 37°C. The MBC was determined according to the method described by Ozturk and Ercisli [26]. In brief, 100 *μ*L from the well corresponding to the MIC value was spread on Mueller–Hinton agar plates. After 18–24 h of incubation at 37°C, the colonies were counted and the concentration that resulted in fewer than 10 colonies was recorded as the MBC value.

### 2.5. FCM and Electron Microscopy (EM) Analyses

The responses of bacteria to final concentrations of 0.5 × MIC, MIC, and 2 × MIC were tested for four strains by adding these concentrations to wells containing bacterial suspensions of 10^8^ CFU/mL^−1^ followed by incubation at 37°C for 12 h. Membrane integrity and bacterial granularity were assessed using FCM and electron microscopy, as described previously [24]. After incubation with the synergistic combination, the cells were washed and resuspended in 950 *μ*L of phosphate-buffered saline (PBS). For FCM analysis, propidium iodide (PI) staining was conducted to assess membrane damage, and bacterial response profiles were measured using a BD FACSCalibur FCM (Becton Dickinson Biosciences, San Jose, CA, United States). Fluorescence intensity was recorded in the FL2 region, a channel designed to detect PI fluorescence (emission at ~585 nm), alongside side scatter (SSC) to evaluate granularity.

The same incubation protocol was followed for SEM, as described in a previous study [27, 28]. After incubation, 2.5% glutaraldehyde was added to the samples, mixed, and incubated at 4°C for 1 h. The samples were then centrifuged at 8000 rpm for 10 min, washed twice with PBS, and resuspended in 950 *μ*L of PBS. SEM was used to examine membrane integrity and bacterial granularity. SEM analyses were performed using the transmission electron microscope JEM-2010 (JEOL Ltd., Germany) and the Quanta 400 FEG (FEI Company, United States) instruments.

### 2.6. Data Analysis

The normality of the data was first evaluated using the Shapiro–Wilk test. All statistical analyses were performed using IBM SPSS Statistics software (Version 29.0.2.0, IBM Corp., Armonk, NY). Data are presented as mean ± standard deviation. Additionally, the populations of viable, membrane-damaged, injured, and dead cells in each sample were analyzed using WinMDI software (Vs 2.9, Scripps Institute, La Jolla, CA).

## 3. Results

### 3.1. Antibacterial Activity Analysis

The antibacterial activity of VFE was tested against both Gram-positive and Gram-negative bacteria. At a concentration of 10 *μ*g disc^−1^, VFE showed no inhibitory effect on the growth of *S. aureus*, *B. subtilis*, or *E. coli*. However, at a concentration of 25 *μ*g disc^−1^, VFE showed antibacterial activity against *S. aureus* and produced an inhibition zone of 6.33 mm. At concentrations of 50 *μ*g/disc or higher, VFE showed an inhibitory activity against both *B. subtilis* and *E. coli* (Table 1). Additionally, the antibacterial activity of VFE against *V. harveyi* was observed at concentrations of 10–1000 *μ*g disc^−1^, with zones of inhibition between 8.66 and 16.33 mm (Table 1).

### 3.2. MIC and MBC Analyses

The MIC, MBC, and MBC/MIC ratios of VFE against the tested pathogenic bacteria are shown in Table 2. VFE showed antibacterial activity against *V. harveyi* with a MIC value of 62.5 *μ*g mL^−1^. The MBC values for the bacteria in the study ranged from 500 to 1000 *μ*g mL^−1^. However, the MBC *for B. subtilis* could not be determined at the tested concentrations of VFE. MIC values of 500 *μ*g mL^−1^ were determined for *E. coli* and *V. harveyi*. The MBC/MIC ratio, which indicates the bactericidal or bacteriostatic potential of the extract, showed that VFE exhibited bactericidal activity against *S. aureus* and *V. harveyi* (Table 2).

### 3.3. FCM Analysis

#### 3.3.1. *B. subtilis*

**Figure 2:**
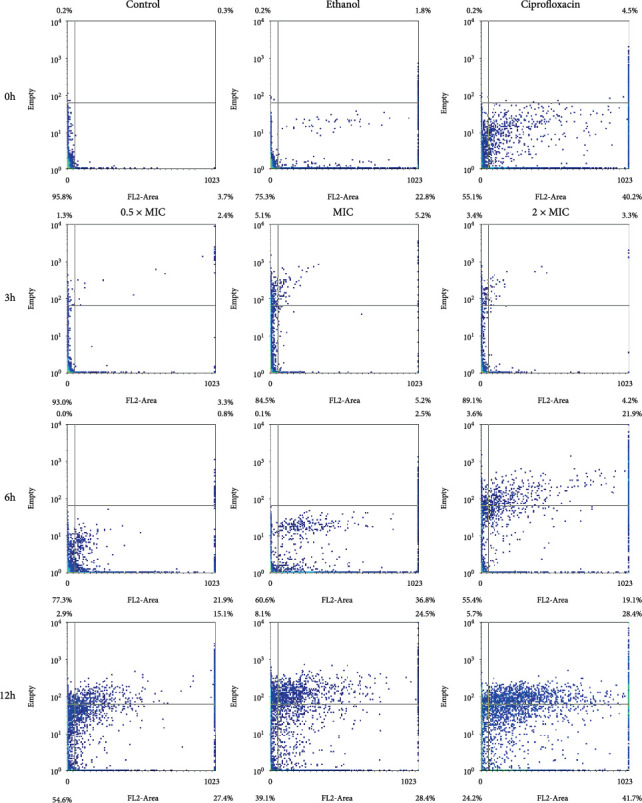
Flow cytometry dot plots for *Bacillus subtilis* treated with 0.5 × MIC, MIC, and 2 × MIC of *Vatica diospyroides* flower extract was incubated at different time points. The regions divided by the lines were interpreted as lower left for viable cells (FL2-negative and SSC negative), lower right for membrane-damaged cells (FL2-positive and SSC-negative), upper left for injured cells (FL2-negative and SSC-positive), and upper right for dead cells (FL2-positive and SSC-positive). PI was used to evaluate bacterial membrane permeability. If loss or damage to the membrane occurred, PI could penetrate the cell and bind with nucleic acids (FL2-positive). Loss of granularity in the injured cell with a breakdown of intracellular components or high complexity could increase the reflection of light (SSC-positive). Each quarter interprets the signals in the density plot accordingly.

The results of the study showed a dose- and time-dependent response of *B. subtilis* to VFE (Table 3). As both the VFE concentration and incubation time increased, the percentage of dead cells also increased. Specifically, the percentage of dead *B. subtilis* cells ranged from 7.0% to 15.1% after 3 h of treatment and increased to 45.4%–75.8% after 12 h. However, the percentage of membrane-damaged or injured cells did not correlate consistently with the VFE dose at any incubation time. This indicates that the extract from the cotyledons induced cell death in *B. subtilis* in a dose-dependent manner. FCM dot plots showed that with increasing concentration and incubation time, cells expanded along the *x*-axis and exhibited increased PI staining, indicating cell membrane disruption (Figure 2).

#### 3.3.2. *Staphylococcus aureus*

**Figure 3:**
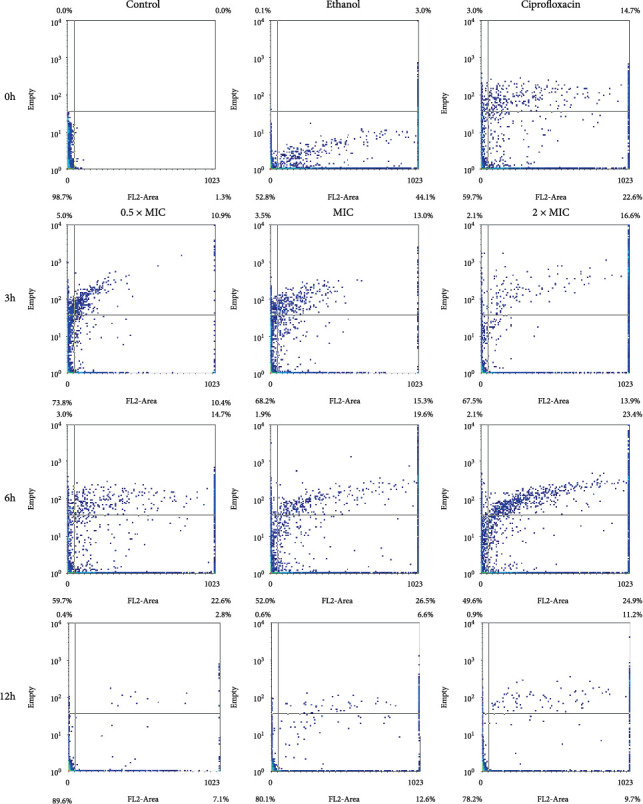
Flow cytometry dot plots for *Staphylococcus aureus* treated with 0.5 × MIC, MIC, and 2 × MIC of *Vatica diospyroides* flower extract were incubated at different time points. The regions divided by the lines were interpreted as lower left for viable cells (FL2-negative and SSC-negative), lower right for membrane-damaged cells (FL2-positive and SSC-negative), upper left for injured cells (FL2-negative and SSC-positive), and upper right for dead cells (FL2-positive and SSC-positive). PI was used to evaluate bacterial membrane permeability. If loss or damage to the membrane occurred, PI could penetrate the cell and bind with nucleic acids (FL2-positive). Loss of granularity in the injured cell with a breakdown of intracellular components or high complexity could increase the reflection of light (SSC-positive). Each quarter interprets the signals in the density plot accordingly.

The results of the response of *S. aureus* to VFE, as shown in Table 4 and Figure 3, show a dose- and time-dependent effect. At all concentrations tested (0.5 × MIC, MIC and 2 × MIC), the graph shows an increase on the *y*-axis and a spread on the *x*-axis during the 3-h incubation period. This pattern indicates abnormalities within the organelles and damage to the cell membrane. As the incubation time increased to 6 h, the proportion of normal cells decreased compared to the 3-h and 12-h time points, indicating a progressive effect of VFE on *S. aureus*.

#### 3.3.3. *Escherichia coli*

**Figure 4:**
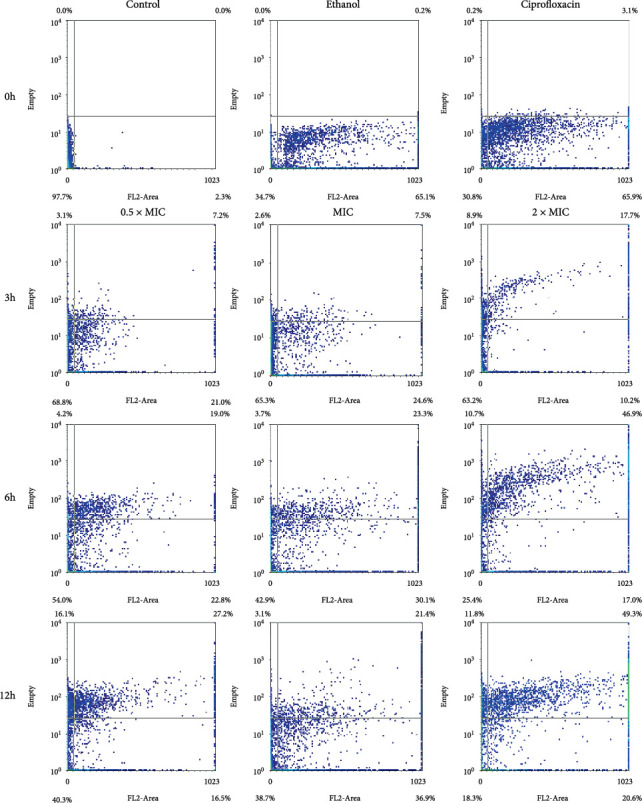
Flow cytometry dot plots for *Escherichia coli* treated with 0.5 × MIC, MIC, and 2 × MIC of *Vatica diospyroides* flower extract were incubated at different time points. The regions divided by the lines were interpreted as lower left for viable cells (FL2-negative and SSC-negative), lower right for membrane-damaged cells (FL2-positive and SSC-negative), upper left for injured cells (FL2-negative and SSC-positive), and upper right for dead cells (FL2-positive and SSC-positive). PI was used to evaluate bacterial membrane permeability. If loss or damage to the membrane occurred, PI could penetrate the cell and bind with nucleic acids (FL2-positive). Loss of granularity in the injured cell with a breakdown of intracellular components or high complexity could increase the reflection of light (SSC-positive). Each quarter interprets the signals in the density plot accordingly.

The study showed that VFE had a similar dose- and time-dependent effect on *E. coli* at concentrations of 0.5 × MIC, MIC, and 2 × MIC as on *B. subtilis* (Table 5). FCM dot plots showed that at 0.5 × MIC, the bacteria exhibited growth along the *y*-axis and partial spreading along the *x*-axis, indicating VFE-induced damage to the organelles and cell membrane of *E. coli*. As the VFE concentration increased, the spreading of cells along the *x*-axis also increased, especially at MIC and 2 × MIC concentrations.  Figure 4 showed that the lower left quadrant, which represents the percentage of live cells, tends to decrease with increasing VFE concentration and incubation time. This indicates a progressive reduction in the number of normal cells with higher extract concentrations and longer exposure times.

#### 3.3.4. *V. harveyi*

**Figure 5:**
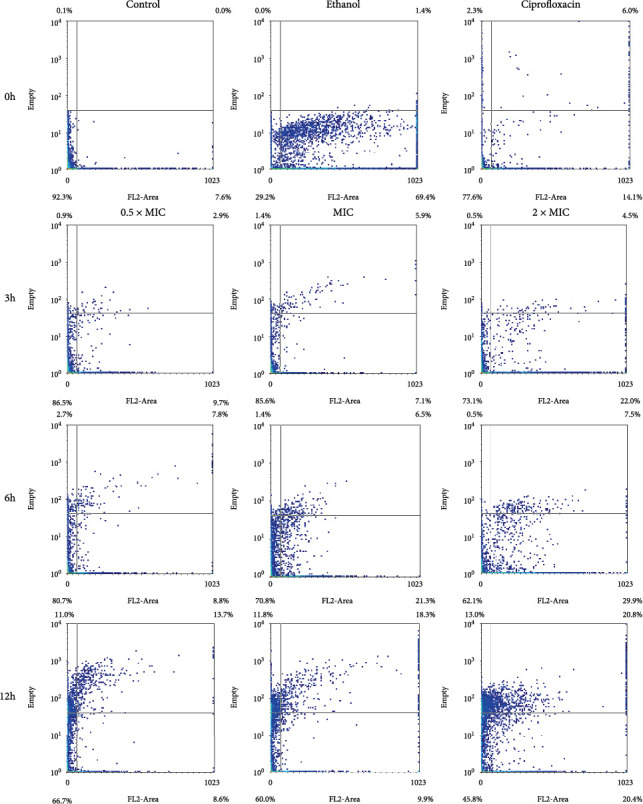
Flow cytometry dot plots for *Vibrio harveyi* treated with 0.5 × MIC, MIC, and 2 × MIC of *Vatica diospyroides* flower extract were incubated at different time points. The regions divided by the lines were interpreted as lower left for viable cells (FL2-negative and SSC negative), lower right for membrane-damaged cells (FL2-positive and SSC-negative), upper left for injured cells (FL2-negative and SSC-positive), and upper right for dead cells (FL2-positive and SSC-positive). PI was used to evaluate bacterial membrane permeability. If loss or damage to the membrane occurred, PI could penetrate the cell and bind with nucleic acids (FL2-positive). Loss of granularity in the injured cell with a breakdown of intracellular components or high complexity could increase the reflection of light (SSC-positive). Each quarter interprets the signals in the density plot accordingly.

The response of *V. harveyi* to VFE is both concentration- and time-dependent.  Figure 5 showed that cell survival decreases when *V. harveyi* is exposed to higher concentrations of VFE over time.  Table 6 showed that the mortality rate increases at higher concentrations between 3 and 6 h but tends to decrease after 12 h. The tests with the 0.5 × MIC, MIC, and 2 × MIC concentrations show that the survival rate of the organism decreases as the VFE concentration increases. The graph shows cell growth along the *y*-axis, with cell proliferation increasing with increasing extract concentration and incubation time. These results indicate that VFE damages the intracellular organelles of *V. harveyi* and ultimately leads to cell death.

### 3.4. SEM Analysis

**Figure 6:**
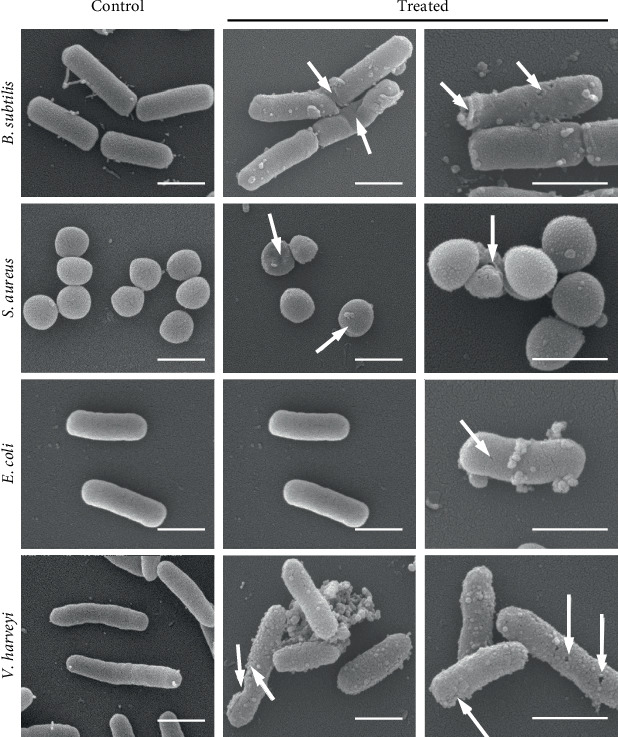
SEM images of *Bacillus subtilis*, *Staphylococcus aureus*, *Escherichia coli*, and *Vibrio harveyi* treated with VFE for 12 h. White arrows indicate the morphological effects of VFE on bacterial cells. SEM analysis was conducted in replicate using two treated samples, with observations made across multiple microscopic fields (scale bars = 1 *μ*m).

SEM analysis of the morphological characteristics of four bacterial strains, as shown in Figure 6, revealed considerable damage to the cell wall. The cells of *B. subtilis* treated with VFE showed remarkable changes compared to the control group, including ruptured cell walls and the appearance of small holes. In *S. aureus*, VFE treatment caused cracks in the cell wall, resulting in broken cells, while the untreated cells showed abnormal shape changes. *E. coli* also showed ruptured cell walls and cracks as well as small holes that changed the appearance of the cells. The results for *V. harveyi* indicated severe cell wall damage, including cracks, fissures, and the formation of numerous small holes in the cell wall.

## 4. Discussion

The search for antibacterial agents derived from natural plant products has become increasingly important, especially in the fight against multidrug-resistant bacteria [6]. Both *B. subtilis* and *E. coli* exhibit multidrug resistance [29, 30], a phenomenon that occurs when bacterial cells accumulate multiple genes that confer resistance to different antibiotics [31]. Additionally, overexpression of multidrug efflux pump genes further exacerbates resistance, as the bacteria can expel different antimicrobial agents, making treatment more difficult [32]. In Thailand, the treatment of infections caused by these resistant bacteria is a major challenge for public health and aquaculture. For example, *V. harveyi*, a common pathogen in the aquatic environment, is associated with both contaminated food and foodborne diseases and poses a significant threat to aquatic animals [33]. In light of these growing challenges, research into alternative antimicrobial strategies is crucial for both food safety and public health, justifying the selection of these bacterial strains for the present study.

Plant metabolites are recognized for their antimicrobial properties, with natural antimicrobials offering significant therapeutic potential by providing effective defense without the health risks linked to synthetic agents [34, 35]. In this study, the antibacterial activity of VFE was investigated using the disk diffusion method. The results showed that VFE exhibited the strongest antibacterial activity against *S. aureus*, producing an inhibition zone of 15.67 mm. Additionally, VFE was effective against bacterial pathogens at MIC ranging from 62.5 to 1000 *μ*g mL^−1^. The primary compounds in *V. diospyroides* are phenolic in nature [14, 15], and these phenolic compounds interact with bacterial cell membranes, causing cell leakage, acidification, and inhibition of biofilm formation [36–38]. Furthermore, VFE contains a high tannin content, which either inactivates enzymes involved in cell wall synthesis or binds directly to the peptidoglycan layer of the bacterial cell wall, increasing bacterial susceptibility to osmotic lysis [39, 40].

Gram-positive bacteria, such as *S. aureus*, are particularly susceptible to phenolic compounds due to the lack of an outer membrane and the exposed peptidoglycan layer on their surface [17]. Polyphenols, as found in many natural compounds, can disrupt the bacterial cell wall by binding to peptidoglycans with their hydroxyl groups and thus weakening the membrane structure [41]. In contrast, Gram-negative bacteria such as *E. coli* have a more complex cell wall structure consisting of an outer membrane, a peptidoglycan layer, and an inner membrane, which makes them less susceptible to the antibacterial effect of phenolic compounds [42]. The high phospholipid content of the outer membrane further contributes to this resistance by limiting the penetration of phenolic compounds. However, the antibacterial effect may occur when hydroxyl groups accumulate in the lipid bilayers, disrupting lipoprotein interactions and increasing membrane permeability [8, 43, 44]. Polyphenols can also damage the integrity of membranes, alter cell morphology, impair metabolism, and cause leakage of cell contents. The destruction of the phospholipid bilayer ultimately leads to cell death by impairing cell division and physiological functions [45, 46]. Within the genus *Vatica*, pericarp and cotyledon extracts of *V. diospyroides* have demonstrated pronounced *in vitro* antibacterial activity against both Gram-positive and Gram-negative bacteria. In disk diffusion assays, the cotyledon extract produced inhibition zones against *B. subtilis*, while pericarp extract produced inhibition zones against both *B. subtilis* and *S. aureus*. Both extracts exhibited MIC values of 1 000 *μ*g mL^−1^ against *B. subtilis and S. aureus*. FCM showed a dose-dependent increase in responder cells of *B. subtilis* treated with cotyledon and pericarp extracts, whereas *S. aureus* responders to pericarp extract increased over time [23]. Other *Vatica* species have exhibited similar potency. For instance, methanolic and acetone extracts of *Vatica odorata* and *Vatica bella* moderately inhibited *S. aureus*, and the methanolic extract of *Vatica bella* also inhibited the dermatophytes *Trichophyton mentagrophytes*, *Trichophyton tonsurans*, and *Microsporum gypseum* [47]. Moreover, the combination of *Phyllanthus emblica*-derived AgNPs with *V. diospyroides* cotyledon extract produced larger inhibition zones against *S. aureus* and *B. subtilis* than AgNPs alone. FCM confirmed a dose-dependent increase in *S. aureus* responders to the combination, characterized by concurrent loss of intracellular components and membrane damage [24]. In this study, VFE showed significant antibacterial activity against *V. harveyi*, which is consistent with previous results on *Vateria copallifera* extract, another member of the Dipterocarpaceae family, which showed high efficacy against *S. aureus* but limited activity against *E. coli* [48]. Phytochemicals exert antimicrobial effects via several mechanisms, including alteration of the physicochemical properties of the plasma membrane, formation of pores, inhibition of DNA gyrase and nucleic acid synthesis, and induction of toxicity via the generation of hydrogen peroxide [8, 49, 50]. Despite these known mechanisms, a complete understanding remains elusive. There is evidence that plant extracts can directly disrupt bacterial membrane structures and possibly form pores, as shown by the time-dependent inhibition observed in some studies [6, 51]. The pore-forming mechanism is supported by FCM data showing increased cell membrane permeability allowing PI to penetrate and bind to DNA, indicating bacterial cell damage [52].

The results of this study showed that VFE induces membrane damage in bacteria, which was confirmed by FCM profiles and electron microscopy images. These analyses showed that VFE impaired the integrity of cell membranes and destroyed intracellular components. These results are consistent with previous studies that have shown that phenolic compounds have different antibacterial effects on different bacterial species depending on their structural configuration [53, 54].

Several limitations must be addressed before VFE can be translated into practical applications in aquaculture. A key limitation of the present study is the lack of phytochemical characterization of the extract. Future research should prioritize comprehensive profiling of VFE's chemical composition using advanced analytical techniques such as high-performance liquid chromatography (HPLC), gas chromatography–mass spectrometry (GC-MS), or liquid chromatography–mass spectrometry (LC-MS/MS). Identifying the major bioactive constituents will support standardization efforts and facilitate the development of advanced delivery systems, such as nanoencapsulation, to enhance the extract's stability, bioavailability, and *in vivo* consistency. Additionally, the potential off-target effects of VFE—including cytotoxicity toward beneficial microbiota and toxicity to nontarget aquatic organisms—remain poorly understood, raising concerns about environmental safety. To address these gaps, future studies should investigate the molecular mechanisms underlying VFE's antibacterial activity, evaluate its in vivo efficacy and ecotoxicological profile in aquaculture models, explore synergistic combinations with conventional antibiotics, optimize formulation technologies, and assess the economic feasibility of large-scale application.

## 5. Conclusion

In conclusion, this study has demonstrated that VFE effectively inhibits the growth of *B. subtilis*, *E. coli*, *S. aureus*, and *V. harveyi* using the well-disk diffusion method. The MIC and MBC values were found to be 250 *μ*g mL^−1^ for *B. subtilis* and *E. coli* and 125 *μ*g mL^−1^ for *S. aureus* and *V. harveyi*. The antibacterial mechanism likely involves denaturation of intracellular structures and the destruction of cell membranes, resulting in bacterial death, as confirmed by electron microscopy. While these results highlight the potential of VFE as an antibacterial agent, the absence of *in vivo* validation and the limited number of bacterial strains tested underscore the need for further research to thoroughly assess the therapeutic potential of *V. diospyroides* flower extract.

## Figures and Tables

**Table 1:** Efficacy of *Vatica diospyroides* flower extract on bacterial strains by disk diffusion method.

**Agents**	**Concentration**	**Inhibition zone diameters (mm)**			
		***S. aureus***	***B. subtilis***	***E. coli***	***V. harveyi***
VFE (*μ*g disc^−1^)	10	0.0 ± 0.0	0.0 ± 0.0	0.0 ± 0.0	8.7 ± 1.2
	25	6.3 ± 1.2	0.0 ± 0.0	0.0 ± 0.0	10.7 ± 0.6
	50	9.3 ± 1.5	8.0 ± 2.0	8.7 ± 3.0	10.3 ± 1.5
	100	10.3 ± 1.5	9.0 ± 3.0	9.0 ± 3.0	11.0 ± 0.0
	200	12.7 ± 3.2	10.0 ± 2.0	11.0 ± 3.6	13.0 ± 1.0
	400	12.7 ± 5.5	10.0 ± 2.0	11.0 ± 3.6	13.0 ± 1.0
	600	13.3 ± 3.5	10.0 ± 1.0	11.3 ± 3.5	15.3 ± 1.5
	800	14.0 ± 1.0	10.3 ± 2.5	11.7 ± 2.5	16.3 ± 1.2
	1000	15.7 ± 4.2	11.7 ± 2.1	11.7 ± 5.1	15.0 ± 4.6

Ciprofloxacin (*μ*g disc^−1^)	30	34.0 ± 0.0	34.0 ± 2.8	29.0 ± 0.0	

Oxytetracycline (*μ*g disc^−1^)	30				30.0 ± 0.0

DMSO (%)	10	0.0 ± 0.0	0.0 ± 0.0	0.0 ± 0.0	0.0 ± 0.0

**Table 2:** MIC, MBC, and MBC/MIC ratio of *Vatica diospyroides* flower extract against bacteria.

**Bacteria**	**Concentration (*μ*g mL** ^**-1**^ **)**		
	**MIC**	**MBC**	**MBC/MIC ratio**
*Staphylococcus aureus*	250	1000	4
*Bacillus subtilis*	1000	—	—
*Escherichia coli*	125	500	4
*Vibrio harveyi*	125	500	4

*Note*: “-“indicates not determined, as no bactericidal endpoint was reached at the highest concentration tested (1000 *μ*g/mL).

**Table 3:** Response pattern of *Bacillus subtilis* when treated with *Vatica diospyroides* flower extract by flow cytometry determination.

**Time (h)**	**Treatment**	**Nonresponder (viable)** ^**1**^	**Injured cells (lost granularity)**	**Membrane-damaged cells**	**Dead cells**	**Total of responder cells**
0	Untreated	95.8	0.2	0.3	3.7	64.2
	Ethanol	75.3	0.2	1.8	22.8	24.7
	Ciprofloxacin	55.1	0.2	4.5	4.2	44.9

3	0.5 × MIC	93.0	1.3	2.4	3.3	7.0
	MIC	84.5	5.1	5.2	5.2	15.1
	2 × MIC	89.1	3.4	3.3	4.2	11.9

6	0.5 × MIC	77.3	0.0	0.8	21.9	22.7
	MIC	60.6	0.1	2.5	36.8	39.4
	2 × MIC	55.4	3.6	21.9	19.1	44.6

12	0.5 × MIC	54.6	2.9	15.1	27.4	45.4
	MIC	39.1	8.1	24.5	28.4	60.9
	2 × MIC	24.2	5.7	28.4	41.7	75.8

^1^Total responders were quantified by Boolean-OR gating of the injured, membrane-damaged, and dead cell populations; %nonresponders = 100% − (events in combined gate ÷ 10,000 total events) × 100.

**Table 4:** Response pattern of *Staphylococcus aureus* when treated with *Vatica diospyroides* flower extract by flow cytometry determination.

**Time (h)**	**Treatment**	**Nonresponder (viable)** ^**1**^	**Injured cells (lost granularity)**	**Membrane-damaged cells**	**Dead cells**	**Total of responder cells**
0	Untreated	98.7	0.0	0.0	1.3	1.3
	Ethanol	52.8	0.1	3.0	44.1	47.2
	Ciprofloxacin	59.7	3.0	14.7	22.6	40.3

3	0.5 × MIC	73.8	5.0	10.9	10.4	26.2
	MIC	68.2	3.5	13.0	15.3	31.8
	2 × MIC	67.5	2.1	16.6	13.9	32.5

6	0.5 × MIC	59.7	3.0	14.7	22.6	40.3
	MIC	52.0	1.9	19.6	26.5	48.0
	2 × MIC	49.6	2.1	23.4	24.9	50.4

12	0.5 × MIC	89.6	0.4	2.8	7.1	10.4
	MIC	80.1	0.6	6.6	12.6	19.9
	2 × MIC	78.2	0.9	11.2	9.7	21.8

^1^Total responders were quantified by Boolean-OR gating of the injured, membrane-damaged, and dead cell populations; %nonresponders = 100% − (events in combined gate ÷ 10,000 total events) × 100.

**Table 5:** Response pattern of *Escherichia coli* when treated with *Vatica diospyroides* flower extract by flow cytometry determination.

**Time (h)**	**Treatment**	**Nonresponder (viable)** ^**1**^	**Injured cells (lost granularity)**	**Membrane-damaged cells**	**Dead cells**	**Total of responder cells**
0	Untreated	97.7	0.0	0.0	2.3	2.3
	Ethanol	34.7	0.0	0.2	65.1	34.9
	Ciprofloxacin	30.8	0.2	3.1	65.9	61.2

3	0.5 × MIC	68.8	3.1	7.2	21.0	31.2
	MIC	65.3	2.6	7.5	24.6	34.7
	2 × MIC	63.2	8.9	17.7	10.2	36.6

6	0.5 × MIC	54.0	4.2	19.0	22.8	46.0
	MIC	42.9	3.7	23.3	30.1	57.1
	2 × MIC	25.4	10.7	46.9	17.0	74.6

12	0.5 × MIC	40.3	16.1	27.2	16.5	59.7
	MIC	38.7	3.1	21.4	36.9	61.3
	2 × MIC	18.3	11.8	49.3	20.6	81.7

^1^Total responders were quantified by Boolean-OR gating of the injured, membrane-damaged, and dead cell populations; %nonresponders = 100% − (events in combined gate ÷ 10,000 total events) × 100.

**Table 6:** Response pattern of *Vibro harveyi* when treated with *Vatica diospyroides* flower extract by flow cytometry determination.

**Time (h)**	**Treatment**	**Nonresponder (viable)** ^**1**^	**Injured cells (lost granularity)**	**Membrane-damaged cells**	**Dead cells**	**Total of responder cells**
0	Untreated	92.3	0.1	0.0	7.6	7.7
	Ethanol	29.2	0.0	1.4	69.4	70.8
	Ciprofloxacin	77.6	2.3	6.0	14.1	22.4

3	0.5 × MIC	86.5	0.9	2.9	9.1	13.5
	MIC	85.6	1.4	5.9	7.1	14.4
	2 × MIC	73.1	0.5	4.5	22.0	26.9

6	0.5 × MIC	80.7	2.7	7.8	8.8	19.3
	MIC	70.8	1.4	6.5	21.3	29.2
	2 × MIC	62.1	0.5	7.5	29.3	37.9

12	0.5 × MIC	66.7	11.0	13.7	8.6	33.3
	MIC	60.0	11.8	18.3	9.9	40.0
	2 × MIC	45.8	13.0	20.8	20.4	54.2

^1^Total responders were quantified by Boolean-OR gating of the injured, membrane-damaged, and dead cell populations; %nonresponders = 100% − (events in combined gate ÷ 10,000 total events) × 100.

## Data Availability

The data that support the finding of this study are available from the corresponding author upon reasonable request.
